# Association Between Prenatal Exposure to Alcohol and Tobacco and Neonatal Brain Activity: Results From the Safe Passage Study

**DOI:** 10.1001/jamanetworkopen.2020.4714

**Published:** 2020-05-12

**Authors:** Lauren C. Shuffrey, Michael M. Myers, Joseph R. Isler, Maristella Lucchini, Ayesha Sania, Nicolò Pini, J. David Nugent, Carmen Condon, Timothy Ochoa, Lucy Brink, Carlie du Plessis, Hein J. Odendaal, Morgan E. Nelson, Christa Friedrich, Jyoti Angal, Amy J. Elliott, Coen Groenewald, Larry Burd, William P. Fifer

**Affiliations:** 1 Department of Psychiatry, Columbia University Irving Medical Center, New York, New York; 2 Division of Developmental Neuroscience, New York State Psychiatric Institute, New York, New York; 3 Department of Pediatrics, Columbia University Irving Medical Center, New York, New York; 4 Department of Obstetrics and Gynaecology, Faculty of Medicine and Health Science, Stellenbosch University, Cape Town, Western Cape, South Africa; 5 Center for Pediatric & Community Research, Avera Research Institute, Sioux Falls, South Dakota; 6 Department of Pediatrics, University of South Dakota School of Medicine, Sioux Falls; 7 Department of Pediatrics, University of North Dakota Medical School, Grand Forks

## Abstract

**Importance:**

Research to date has not determined a safe level of alcohol or tobacco use during pregnancy. Electroencephalography (EEG) is a noninvasive measure of cortical function that has previously been used to examine effects of in utero exposures and associations with neurodevelopment.

**Objective:**

To examine the association of prenatal exposure to alcohol (PAE) and tobacco smoking (PTE) with brain activity in newborns.

**Design, Setting, and Participants:**

This prospective cohort study enrolled mother-newborn dyads from December 2011 through August 2015, with data analyzed from June 2018 through June 2019. Pregnant women were recruited from clinical sites in Cape Town, South Africa, and the Northern Plains region of the US. Participants were a subset of newborns enrolled in the Safe Passage Study. Exclusions included birth at less than 37 or more than 41 weeks’ gestation, multiple birth, or maternal use of psychiatric medication during pregnancy.

**Exposures:**

PAE and PTE groups were determined by cluster analysis.

**Main Outcomes and Measures:**

Analyses of covariance were run on EEG spectral power at 12 scalp locations across the frequency spectrum from 1 to 45 Hz in 3-Hz bins by sleep state.

**Results:**

The final sample consisted of 1739 newborns (median [interquartile range] gestational age at birth, 39.29 [1.57] weeks; 886 [50.9%] were female; median [interquartile range] newborn age at assessment, 48.53 [44.96] hours). Newborns whose mothers were in the low continuous (95% CI, −0.379 to −0.031; *P* < .05; 95% CI, −0.379 to −0.045; *P* < .05), quit (95% CI, −0.419 to −0.127; *P* < .001; 95% CI, −0.398 to −0.106; *P* < .005), and moderate or high continuous (95% CI, −0.430 to −0.124; *P* < .001; 95% CI, −0.420 to −0.119; *P* < .005) PAE clusters had increased 4- to 6-Hz and 7- to 9-Hz left-temporal EEG power. Newborns with moderate or high continuous PTE had decreased 19- to 21-Hz (95% CI, 0.034 to 0.327; *P* < .05) and 22- to 24-Hz (95% CI, 0.022 to 0.316; *P* < .05) right-central EEG compared with newborns with no PTE. Newborns with moderate or high continuous PTE had significantly decreased 22- to 36-Hz right-central EEG power compared with the quit smoking group (22-24 Hz, 95% CI, 0.001 to 0.579; *P* < .05; 25-27 Hz, 95% CI, 0.008 to 0.586; *P* < .05; 28-30 Hz, 95% CI, 0.028 to 0.607; *P* < .05; 31-33 Hz, 95% CI, 0.038 to 0.617; *P* < .05; 34-36 Hz, 95% CI, 0.057 to 0.636; *P* < .05).

**Conclusions and Relevance:**

These findings suggest that even low levels of PAE or PTE are associated with changes in offspring brain development.

## Key Points

**Question:**

Are prenatal alcohol exposure (PAE) and prenatal tobacco exposure (PTE) associated with brain activity in newborns during natural sleep?

**Findings:**

In this cohort study of 1739 mother-newborn dyads, patterns of PAE and PTE were associated with neonatal electroencephalography power. PAE was associated with increased low-frequency brain activity at temporal electrode sites, whereas moderate or high continuous PTE was associated with decreased high-frequency brain activity at central electrode sites.

**Meaning:**

The findings suggest that any level of PAE or PTE is associated with newborn brain development, reaffirming the public health message that research has not yet determined a safe level of alcohol or tobacco use during pregnancy.

## Teaser

This cohort study uses electroencephalography to examine the association of prenatal exposure to alcohol and tobacco smoking with brain activity in newborns.

## Introduction

Negative long-term effects of excessive prenatal alcohol exposure (PAE) and prenatal tobacco exposure (PTE) on risk for multiple adverse outcomes have been well established. PAE is the leading cause of preventable intellectual disability, and smoking during pregnancy is one of the most modifiable causes of perinatal morbidity and mortality.^1,2^ Understanding the associations of quantity, timing, and various combinations of in utero alcohol and smoking exposures with early brain function could help identify mechanisms that underlie adverse long-term neurobehavioral outcomes.

Assessing neonatal brain activity through electroencephalography (EEG) provides a means of examining potential associations of PAE and PTE with brain activity in the immediate postnatal period. EEG is a noninvasive, sensitive measure of brain activity reflecting electrical activity generated by spatially aligned postsynaptic potentials in cortical pyramidal cells owing to their orientation in relation to the cortex.^3,4,5,6^ EEG spectral power is a measure of the amplitude of the EEG signal from peak to peak across a specified length of time.^3,4,5,6^ As children age, there is a developmental decrease in low-frequency brain oscillations, delta (δ) and theta (θ), starting around 4 years of age and a developmental increase in high-frequency brain activity beta (β) and gamma (γ).^3,4,5,6^ Longitudinal studies of alpha (α) power during infancy suggest a developmental increase in 6- to 9-Hz activity with central alpha peaking around 2 years of age.^7^ Although less is known regarding the development of EEG power during the neonatal period, developmental changes in oscillatory activity are postulated to reflect decreases in synaptic density that underlie neural pruning to increase functional specialization.^7,8^ EEG power in newborns has been shown to predict developmental outcomes at later ages when controlling for gestational age at birth and sleep state.^9,10,11,12,13^ Although there is significant heterogeneity in prior studies, relative to neurotypical populations, infants at risk for developmental disorders often exhibit atypical developmental trajectories in neural oscillations, such as increased delta and theta or decreased beta or gamma power.^14,15,16,17,18,19,20,21,22^

To our knowledge, only 3 prior studies have examined associations between PAE or PTE and infant brain function by using EEG.^23,24,25^ Although these studies were restricted by small sample size and only examined high levels of PAE, each reported increased EEG power in infants with PAE, described as hypersynchronous EEG.^23,24,25^ Increased EEG power in infants of alcoholic mothers was highest in rapid eye movement (REM) sleep, where power was approximately 200% greater than controls.^23^ This finding was further supported by preclinical studies demonstrating increased hippocampal theta rhythm activity in rats with PAE.^26^ Increased EEG power in infants with PAE is likely not attributable to alcohol withdrawal syndrome given that neurophysiologic differences persist 4 to 6 weeks postnatally.^25^ More recently, a study has identified hypersynchrony in magnetoencephalography spectral power in awake 6-month-old infants with low to moderate PAE compared with controls.^27^ The association was persistent across all frequency bands and was most prominent in left anterior and posterior temporal regions.^27^ Although 1 study found no differences in EEG power in infants with PTE compared with controls with minimal or no exposure,^24^ more recent data suggest neurophysiologic sensitivity to prenatal nicotine exposure (PNE). An EEG/event-related potential study examined the auditory K-complex in infants 3 to 5 months old with PNE and found reduced delta power compared with unexposed infants in non-REM sleep.^28^ However, a study of PNE on sleep/wake ontogenesis in neonatal rats demonstrated increased delta and theta Hz activity in REM sleep.^29^ Recent evidence also suggests that PAE and PNE combined induce oxidative stress and increase monoamine oxidase activity and caspase expression in the cerebellum.^30^ These biochemical aberrations from dual PAE and PNE suggest potential compounding neurotoxic effects on the developing brain.

The current prospective study of 1739 newborns examines associations of PAE and PTE with neonatal brain activity measured via EEG spectral power. To examine several different patterns of PAE and PTE, we have used cluster analysis to carefully characterize drinking and smoking patterns. Based on prior clinical and preclinical studies, we hypothesized that dual prenatal exposure to alcohol and smoking would be associated with increased low-frequency EEG power and decreased high-frequency EEG power in neonates.

## Methods

### Participants

Participants were a subset of neonates with neonatal EEG data enrolled in the Safe Passage Study conducted by the Prenatal Alcohol and SIDS and Stillbirth (PASS) Network, a multicenter study investigating the role of prenatal exposure to alcohol and smoking in risk for multiple adverse outcomes.^31^ Mother-newborn dyads were enrolled from December 2011 through August 2015. Data were analyzed between June 2018 through June 2019. Participants were excluded from the present analysis on the basis of multiple birth, birth before 37 weeks’ gestation or after 41 weeks’ gestation, or prenatal exposure to any psychiatric medications at any point during pregnancy (selective serotonin reuptake inhibitors, antidepressants, classic antipsychotics, atypical antipsychotics, mood stabilizers, stimulants, anxiolytics, or anticonvulsants). Written informed consent to record neonate brain activity using EEG was obtained as part of the consent for the main study. Ethical approval was obtained from Stellenbosch University, Sanford Health, the Indian Health Service, and New York State Psychiatric Institute.

### Self-reported Exposure Measures

The procedures used to obtain detailed information about quantity and timing of PAE and PTE have previously been described by the PASS Network.^31,32^ In brief, information regarding PAE was acquired using a modified 30-day Timeline Followback interview^31,32^ in which women self-reported their daily alcohol consumption for their last drinking day and 30 days prior up to 4 times during pregnancy. Detailed information was acquired regarding drink sharing, the type and brand of alcohol, container size, and duration of drinking to estimate the amount of alcohol consumed as accurately as possible.^31,32^ This information was used to calculate an estimate of total grams of alcohol consumed per day for each day during pregnancy. Agreement between the Safe Passage Study Timeline Followback interview and neonate meconium alcohol marker ethyl glucuronide demonstrated 82% sensitivity (95% CI, 71.6%-92.0%) and 75% specificity (95% CI, 63.2%-86.8%) between PAE and ethyl glucuronide.^33^ PTE information was also obtained up to 4 times during pregnancy in which women reported their smoking habits for their last reported smoking day and 30 days prior. Women indicated how often and the quantity they smoked tobacco cigarettes on an average smoking day.^31,34^ These estimates were used to calculate average cigarettes smoked per week for each week of pregnancy.

### Neonatal EEG Acquisition and Processing

EEG data were acquired during the newborns’ natural sleep using a hybrid system of a 28-lead high-impedance electrode net (Electrical Geodesics) and a miniature amplifier recording device (ATES). EEG data collection and processing procedures were previously described (eMethods in the Supplement).^35^

### Statistical Analyses

#### Missing Data Imputation

We imputed missing daily alcohol and weekly smoking data by a nonparametric machine learning algorithm called the k-nearest neighbor approach.^36,37,38^ More detail is available in the eMethods in the Supplement.

#### Alcohol and Smoking Cluster Analysis

To discern associations between different patterns of PAE and PTE during pregnancy on newborn brain activity, we implemented cluster analysis to characterize multiple patterns of maternal drinking and smoking behaviors (eMethods in the Supplement).^38^ In the present analysis, we collapsed the PASS alcohol and smoking cluster groups^38^ to create a 4-level PAE variable (no alcohol, low continuous alcohol, quit early alcohol, moderate or high continuous alcohol) and a 3-level PTE variable (no smoking, low continuous or quit early smoking, moderate or high continuous smoking) (Table 1). More detail is available in the eMethods and eTables 2 and 3 in the Supplement.

**Table 1.** Cross Tabulation of Each Possible Alcohol Cluster Group and Smoking Cluster Group Combination

Alcohol cluster group	Smoking cluster group			
	No Smoking	Low continuous or quit early smoking	Moderate or high continuous smoking	Total
No alcohol	482	203	93	778
Low continuous alcohol	54	75	49	178
Quit early alcohol	272	162	48	482
Moderate or high continuous alcohol	51	140	110	301
Total	859	580	300	1739

#### Computing EEG Power

Absolute EEG power, representing the square of EEG magnitude, was calculated for 12 scalp regions (left and right: frontal-polar, frontal, central, parietal, temporal, and occipital) for 15 frequencies in 3-Hz–wide frequency bins from 1 Hz to 45 Hz separately for active sleep (AS) and quiet sleep (QS) (eTable 1 in the Supplement). Owing to significant differences in the mean (SD) postnatal age at assessment between clinical sites (South Africa: 60.73 [21.72] hours; Northern Plains: 24.68 [9.56] hours; *P* < .001), the standardized residual of EEG power, after adjusting for postnatal age assessment within clinical site, was used for all subsequent analyses.

#### Statistical Analyses of EEG Power

Analyses for examining the association of PAE and PTE with EEG power controlled for sex, gestational age at birth, clinical site, and recreational drug exposure. Analyses of covariance were run by sleep state for each frequency bin and scalp region to examine the main effect of alcohol, the main effect of smoking, and an interaction term between alcohol and smoking, resulting in 180 statistical comparisons. Hypothesis tests were 2-sided. A 10% false discovery rate (FDR) correction was implemented to correct for multiple comparisons using the Benjamini-Hochberg procedure.^39^ In the presence of a significant main effect, all pairwise comparisons were run where we reported 95% CIs for the difference and pairwise *P* values. Statistical analyses were performed with R, version 3.6.1 (R Studio) and SPSS, version 26 (IBM Corp). This study followed the Strengthening the Reporting of Observational Studies in Epidemiology (STROBE) reporting guideline.

## Results

### Summary Demographic Information

The final sample consisted of a subset of 1739 term-age neonates from the Safe Passage Study with available maternal prenatal exposure information and neonatal EEG data (886 [50.9%] were female) (eMethods in the Supplement). The median (interquartile range) gestational age at birth was 39.29 (1.57) weeks, and newborns were median (interquartile range) 48.53 (44.96) hours postnatal at the EEG study (Table 2).

**Table 2.** Study Participant Demographic Information

Characteristic	No. (%)		
	All clinical sites	Northern Plains, US (n = 481 [27.7%])	Western Cape, South Africa (n = 1258 [72.3%])
Gestational age at birth, median (IQR), wk	39.29 (1.57)	39.29 (1.29)	39.14 (1.57)
Newborn age at study assessment, median (IQR), postnatal h	48.53 (44.96)	24.35 (12.20)	61.00 (36.67)
Sex			
Female	886 (50.9)	248 (51.6)	638 (50.7)
Male	853 (49.1)	233 (48.4)	620 (49.3)
Race/ethnicity			
American Indian or Alaska Native	142 (8.2)	142 (29.5)	0 (0)
Mixed race	1256 (72.2)	0	1256 (99.9)
White	277 (15.9)	277 (57.6)	0
Other	64 (3.7)	62 (12.9)	2 (0.1)
Delivery mode			
Vaginal			
Spontaneous	1440 (82.8)	353 (73.3)	1087 (86.4)
Operative	60 (3.5)	21 (4.4)	39 (3.1)
Cesarean	239 (13.7)	107 (22.2)	132 (10.5)
Maternal characteristics			
Age at delivery, median (IQR), y	25.0 (9.0)	28.0 (8.0)	24.0 (9.0)
Married	989 (56.9)	383 (79.7)	606 (48.1)
Education level			
Primary school	86 (4.9)	4 (0.8)	82 (6.5)
Some high school	912 (52.4)	68 (14.1)	844 (67.1)
Completed high school	371 (21.3)	89 (18.5)	282 (22.4)
Beyond high school	370 (21.3)	320 (66.5)	50 (3.9)

Abbreviation: IQR, interquartile range.

### Main Effect of Alcohol on EEG Power in Active Sleep

**Figure 1.**
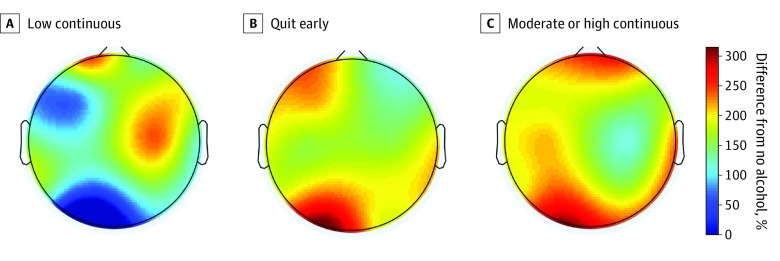
Main Effect of Alcohol on Electroencephalography Power Main effect of alcohol at 4-6 Hz (theta) on newborn electroencephalography power representing the percent difference in power compared with no alcohol based on estimated marginal means.

After FDR correction for multiple comparisons, there was a significant main effect of alcohol on right-temporal 4- to 6-Hz, left-temporal 4- to 6-Hz, and left-temporal 7- to 9-Hz theta and (infant) alpha EEG power (Table 3; Figure 1). The Benjamini-Hochberg critical value for passing *P* values was *P* = .001. Pairwise comparisons revealed that neonates with low continuous PAE did not significantly differ from those with no PAE in right-temporal theta EEG power (*P* > .05; eTables 4 and 5 in the Supplement). However, neonates whose mothers were in the quit and moderate or high continuous PAE clusters had significantly increased right-temporal theta EEG power compared with neonates with no PAE (quit: 95% CI, −0.390 to −0.099; *P* < .001; moderate or high continuous: 95% CI, −0.431 to −0.125; *P* < .001; eTables 4 and 5 in the Supplement). Additionally, infants of mothers in the low continuous, quit, and moderate or high continuous PAE groups all had significantly higher left-temporal theta and alpha EEG power compared with infants with no PAE (4-6 Hz, low continuous: 95% CI, −0.379 to −0.031; *P* < .05; quit: 95% CI, −0.419 to −0.127; *P* < .001; moderate or high continuous: 95% CI, −0.430 to −0.124; *P* < .001; 7-9 Hz, low continuous: 95% CI, −0.379 to −0.045; *P* < .05, quit: 95% CI, −0.398 to −0.106; *P* < .005; moderate or high continuous: 95% CI, −0.420 to −0.119; *P* < .005; eTables 4 and 5 in the Supplement). The largest contrasts in temporal theta and alpha EEG power were observed between infants with no PAE and infants with moderate or high continuous PAE (Figure 1).

**Table 3.** Main Effect of Alcohol and Smoking on Newborn Electroencephalography Power in Active Sleep

Main effect	Brain region	Frequency bin	*F* statistic	*P* value	Partial eta^2^	Observed power
Alcohol	Right temporal	4-6 Hz (theta)	5.82	.001	0.010	0.95
	Left temporal	4-6 Hz (theta)	6.64	<.001	0.012	0.97
		7-9 Hz (infant alpha)	6.16	<.001	0.011	0.96
Smoking	Right central	19-21 Hz (beta)	5.81	.003	0.007	0.87
		22-24 Hz (beta)	6.11	.002	0.008	0.88
		25-27 Hz (low gamma)	5.13	.006	0.006	0.82
		28-30 Hz (low gamma)	6.77	.001	0.008	0.91
		31-33 Hz (low gamma)	6.89	.001	0.009	0.92
		34-36 Hz (low gamma)	6.60	.001	0.008	0.91
	Right parietal	28-30 Hz (low gamma)	5.35	.005	0.006	0.84
		31-33 Hz (low gamma)	5.78	.003	0.007	0.86
		34-36 Hz (low gamma)	5.84	.003	0.007	0.87
		37-39 Hz (gamma)	5.40	.005	0.007	0.84
		43-45 Hz (gamma)	5.83	.003	0.007	0.87

### Main Effect of Smoking on EEG Power in Active Sleep

**Figure 2.**
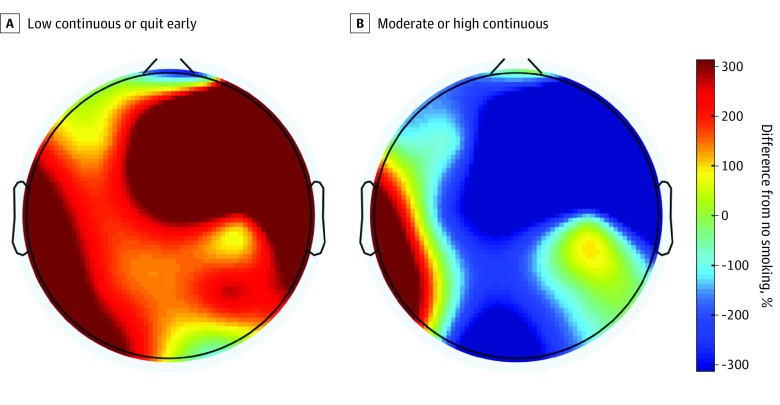
Main Effect of Smoking on Electroencephalography Power Main effect of smoking at 19-21 Hz (beta) on newborn electroencephalography power representing the percent difference in power compared with no smoking based on estimated marginal means.

After FDR correction for multiple comparisons, there were also significant main effects of PTE. The Benjamini-Hochberg critical value for passing *P* values was *P* = .006. However, in contrast to PAE, these associations were seen at higher EEG frequencies, specifically in the right-central region (19-36 Hz) and right-parietal region (28-39 Hz; 43-45 Hz) (Table 3; Figure 2). Infants of mothers in the low continuous or quit PTE cluster had significantly increased right-central beta and low gamma EEG power compared with infants with no PTE (19-21 Hz, 95% CI, −0.306 to −0.021; *P* < .05; 22-24 Hz, 95% CI, −0.305 to −0.019; *P* < .05; 25-27 Hz, 95% CI, −0.321 to −0.034; *P* < .05; 28-30 Hz, 95% CI, −0.349 to −0.063; *P* < .01; 31-33 Hz, 95% CI, −0.351 to −0.064; *P* < .01; 34-36 Hz, 95% CI, −0.356 to −0.070; *P* < .01) (eTables 6 and 8 in the Supplement). Infants with mothers in the low continuous or quit PTE cluster also had significantly increased right-parietal low gamma and gamma EEG power compared with infants with no PTE (28-30 Hz, 95% CI, −0.361 to −0.076; *P* < .01; 31-33 Hz, 95% CI, −0.371 to −0.086; *P* < .01; 34-36 Hz, 95% CI, −0.374 to −0.09; *P* < .01; 37-39 Hz, 95% CI, −0.365 to −0.08; *P* < .01; and 43-45 Hz, 95% CI, −0.379 to −0.094; *P* < .01) (eTables 9-11 in the Supplement). However, the largest contrasts were between infants with moderate or high continuous PTE who had significantly decreased right-central (19-33 Hz) and right-parietal (28-39 Hz; 43-45 Hz) beta, low gamma, and gamma EEG power compared with the low continuous or quit PTE cluster (eTables 6-11 in the Supplement).

Because of the unexpected finding in the low continuous or quit smoking group, post hoc pairwise comparisons were used to explicate the association between quitting smoking (n = 66) and newborn brain activity using a 4-level smoking variable and additionally controlling for PAE (eTable 12 in the Supplement). There was no significant main effect of quitting smoking on right-central beta EEG power (19-21 Hz) or right-parietal low gamma or gamma EEG power (28-45 Hz) (eTables 14-17 in the Supplement; *P* > .05 for all). However, the quit smoking group had significantly increased right-central beta and low gamma EEG power compared with the moderate or high continuous PTE group (22-24 Hz, 95% CI, 0.001 to 0.579; *P* < .05; 25-27 Hz, 95% CI, 0.008 to 0.586; *P* < .05; 28-30 Hz, 95% CI, 0.028 to 0.607; *P* < .05; 31-33 Hz, 95% CI, 0.038 to 0.617; *P* < .05; 34-36 Hz, 95% CI, 0.057 to 0.636; *P* < .05; eTables 13-15 in the Supplement). Pairwise comparisons also revealed a significant decrease in right-central beta EEG power for moderate or high continuous PTE compared with no PTE (19-21 Hz, 95% CI, 0.034 to 0.327; *P* < .05; 22-24 Hz, 95% CI, 0.022 to 0.316; *P* < .05) (eTables 13 and 14 in the Supplement). There were no significant differences between moderate or high continuous PTE or low continuous PTE compared with no PTE for 25- to 36-Hz right-central or 37- to 45-Hz right-parietal EEG power (eTables 15-18 in the Supplement; *P* > .05 for all).

### Associations of Alcohol and Smoking With EEG Power in Active and Quiet Sleep

There were no statistically significant associations between alcohol and smoking and EEG power in AS. Sample size in QS was reduced from 1739 in AS to 1201. After FDR correction, there were no statistically significant main effects or significant associations between PAE and PTE and EEG power in QS.

## Discussion

To our knowledge, the present report is the largest study to date to investigate associations between PAE and PTE and brain activity in newborns. Through careful characterization of maternal drinking and smoking behaviors using cluster analysis, we demonstrated that PAE and PTE are associated with distinct infant brain activity patterns in the AS state. PAE was associated with increased theta and alpha EEG power in a dose-dependent manner in which infants with moderate or high continuous PAE had the most significant increase compared with infants with no PAE. Specifically, low continuous PAE was associated with a 147% increase in theta power at left-temporal electrode sites compared with no PAE, whereas moderate or high continuous PAE was associated with a 199% increase compared with no PAE. We found divergent associations from moderate or high continuous PTE and low continuous or quitting smoking with an unexpected increased in beta and gamma EEG power in the low continuous or quit smoking group at right-central and right-parietal electrode sites.

Although there was no statistical interaction between PAE and PTE on EEG power, their independent associations with EEG power suggest abnormal maturation of cortical networks. To our knowledge, we are the first to report associations between PTE and neonatal EEG power in human infants. However, several preclinical studies have demonstrated a bimodal response to nicotine suggesting smoking may affect biological systems through multiple mechanisms.^40,41,42,43^ EEG does not elucidate the mechanisms of these associations; however, we hypothesize they could potentially result from either functional alterations in neuronal differentiation,^44,45,46^ fetal hypoxia as a result of decreased uterine perfusion and vasoconstriction from adrenergic discharge,^47^ or increased carboxyhemoglobin^48^ at a critical window in development.

Our PAE findings are in partial agreement with prior reports that demonstrated increased EEG power in infants of alcoholic mothers, described as hypersynchronous EEG,^23,24,25^ and with the recent report of increased magnetoencephalography power in 6-month-old infants with low to moderate PAE.^27^ However, we are the first to report associations between PAE and brain activity even in infants with low continuous PAE and in infants whose mothers quit drinking before the second trimester. This finding has significant public health relevance in the context of media reports on the lack of perinatal effects from light drinking during pregnancy. The consistent finding of increased EEG power from PAE may reflect an imbalance in the excitatory glutamate to inhibitory γ-aminobutyric acid ratio resulting in weakened neural inhibition, increased neural excitation, or aberrant neuronal differentiation.^49^ Evidence from in vitro studies has demonstrated that PAE results in increased amplitude and duration of excitatory hippocampal pyramidal cellular activity.^50^

Although we have not yet determined the association between changes in brain activity at birth from PAE and subsequent cognitive or behavioral outcomes, our present findings from a diverse, multinational cohort are especially important in the context of recent reports suggesting either no effect or a protective effect from low to moderate PAE on birth, academic, cognitive, and attentional outcomes in women from high socioeconomic households.^51,52,53^ Because women with low levels of education or advanced maternal age who consume alcohol during pregnancy are at the greatest risk for having a child with fetal alcohol spectrum disorders,^54^ it is important to assess brain function at birth independent of potential modifying factors in an enriched postnatal environment.^54^ Taken together, our findings suggest that any level of PAE or PTE has robust associations with newborn brain activity, reaffirming the public health message that research has not yet determined a safe level of alcohol or tobacco use during pregnancy.

### Limitations

Although we attempted to accurately estimate drinking and smoking behaviors, it is possible there could be underreporting or overreporting of PAE or PTE. The present study only reports the associations of PAE and PTE in term-age infants, which may vary in preterm birth, and we measured EEG at only 1 time point. In future reports, we plan to examine additional exposures and long-term neurodevelopmental outcomes.

## Conclusions

Examining neonatal EEG may prove to be a reliable proximal marker of the potential downstream associations of PAE and PTE with neurodevelopment. We hope these data can elucidate potential mechanisms underlying risk for adverse outcomes.

## Notes

Supplement. **eMethods.** **eTable 1.** EEG Frequency Bin to Frequency Name Reference **eTable 2.** Alcohol Clusters **eTable 3.** Smoking Clusters **eTable 4.** Estimated marginal means for the main effect of alcohol on temporal Theta and Alpha EEG power in active sleep **eTable 5.** Pairwise comparisons for the main effect of alcohol compared to no prenatal exposure to alcohol **eTable 6.** Estimated marginal means for the main effect of smoking on right central EEG power in active sleep **eTable 7.** Pairwise comparisons for the effect of smoking on Beta (19 – 24 Hz) right central EEG power in active sleep **eTable 8.** Pairwise comparisons for the effect of smoking on low Gamma (25 – 37 Hz) right central EEG power in active sleep **eTable 9.** Estimated marginal means for the main effect of smoking on right parietal EEG power in active sleep **eTable 10.** Pairwise comparisons for the effect of smoking on low Gamma (28 – 36 Hz) right parietal EEG power in active sleep **eTable 11.** Pairwise comparisons for the effect of smoking on Gamma (37 – 39; 43 – 45 Hz) right parietal EEG power in active sleep **eTable 12.** 4-level Collapsed Smoking Cluster **eTable 13.** Estimated marginal means for the main effect of smoking using a 4-level smoking variable on right central EEG power in active sleep **eTable 14.** Pairwise comparisons for the effect of smoking on Beta (19 - 24 Hz) right central EEG power in active sleep **eTable 15.** Pairwise comparisons for the effect of smoking on low Gamma (25 - 36 Hz) right central EEG power in active sleep **eTable 16.** Estimated marginal means for the main effect of smoking using a 4-level smoking variable on right parietal EEG power in active sleep **eTable 17.** Pairwise comparisons for the effect of smoking on low Gamma (28 – 36) right parietal EEG power in active sleep **eTable 18.** Pairwise comparisons for the effect of smoking on Gamma (37 – 39; 43 – 25 Hz) right parietal EEG power in active sleep **eReferences**
