# Supplementary material for: Association Between Prenatal Exposure to Alcohol and Tobacco and Neonatal Brain Activity: Results From the Safe Passage Study
Source: JAMA Netw Open. 2020 May 12;3(5):e204714. doi: 10.1001/jamanetworkopen.2020.4714 (PMC7218492; doi:10.1001/jamanetworkopen.2020.4714)
Supplement: Supplement. — eMethods. eTable 1. EEG Frequency Bin to Frequency Name Reference eTable 2. Alcohol Clusters eTable 3. Smoking Clusters eTable 4. Estimated marginal means for the main effect of alcohol on temporal Theta and Alpha EEG power in active sleep eTable 5. Pairwise comparisons for the main effect of alcohol compared to no prenatal exposure to alcohol eTable 6. Estimated marginal means for the main effect of smoking on right central EEG power in active sleep eTable 7. Pairwise comparisons for the effect of smoking on Beta (19 – 24 Hz) right central EEG power in active sleep eTable 8. Pairwise comparisons for the effect of smoking on low Gamma (25 – 37 Hz) right central EEG power in active sleep eTable 9. Estimated marginal means for the main effect of smoking on right parietal EEG power in active sleep eTable 10. Pairwise comparisons for the effect of smoking on low Gamma (28 – 36 Hz) right parietal EEG power in active sleep eTable 11. Pairwise comparisons for the effect of smoking on Gamma (37 – 39; 43 – 45 Hz) right parietal EEG power in active sleep eTable 12. 4-level Collapsed Smoking Cluster eTable 13. Estimated marginal means for the main effect of smoking using a 4-level smoking variable on right central EEG power in active sleep eTable 14. Pairwise comparisons for the effect of smoking on Beta (19 - 24 Hz) right central EEG power in active sleep eTable 15. Pairwise comparisons for the effect of smoking on low Gamma (25 - 36 Hz) right central EEG power in active sleep eTable 16. Estimated marginal means for the main effect of smoking using a 4-level smoking variable on right parietal EEG power in active sleep eTable 17. Pairwise comparisons for the effect of smoking on low Gamma (28 – 36) right parietal EEG power in active sleep eTable 18. Pairwise comparisons for the effect of smoking on Gamma (37 – 39; 43 – 25 Hz) right parietal EEG power in active sleep eReferences [file jamanetwopen-3-e204714-s001.pdf]

Supplementary Online Content

Shuffrey LC, Myers MM, Isler JR, et al; PASS Network. Association between prenatal exposure to alcohol and tobacco and neonatal brain activity: results from the Safe Passage Study. *JAMA Netw Open*. 2020;3(5):e204714. doi:10.1001/jamanetworkopen.2020.4714

eMethods.
eTable 1. EEG Frequency Bin to Frequency Name Reference
eTable 2. Alcohol Clusters
eTable 3. Smoking Clusters
eTable 4. Estimated marginal means for the main effect of alcohol on temporal Theta and Alpha EEG power in active sleep
eTable 5. Pairwise comparisons for the main effect of alcohol compared to no prenatal exposure to alcohol
eTable 6. Estimated marginal means for the main effect of smoking on right central EEG power in active sleep
eTable 7. Pairwise comparisons for the effect of smoking on Beta (19 – 24 Hz) right central EEG power in active sleep
eTable 8. Pairwise comparisons for the effect of smoking on low Gamma (25 – 37 Hz) right central EEG power in active sleep
eTable 9. Estimated marginal means for the main effect of smoking on right parietal EEG power in active sleep
eTable 10. Pairwise comparisons for the effect of smoking on low Gamma (28 – 36 Hz) right parietal EEG power in active sleep
eTable 11. Pairwise comparisons for the effect of smoking on Gamma (37 – 39; 43 – 45 Hz) right parietal EEG power in active sleep
eTable 12. 4-level Collapsed Smoking Cluster
eTable 13. Estimated marginal means for the main effect of smoking using a 4-level smoking variable on right central EEG power in active sleep
eTable 14. Pairwise comparisons for the effect of smoking on Beta (19 - 24 Hz) right central EEG power in active sleep
eTable 15. Pairwise comparisons for the effect of smoking on low Gamma (25 - 36 Hz) right central EEG power in active sleep
eTable 16. Estimated marginal means for the main effect of smoking using a 4-level smoking variable on right parietal EEG power in active sleep
eTable 17. Pairwise comparisons for the effect of smoking on low Gamma (28 – 36) right parietal EEG power in active sleep
eTable 18. Pairwise comparisons for the effect of smoking on Gamma (37 – 39; 43 – 25 Hz) right parietal EEG power in active sleep
eReferences

This supplementary material has been provided by the authors to give readers additional information about their work.

© 2020 Shuffrey LC et al. *JAMA Network Open.*

eMethods.

Neonatal EEG Acquisition and Processing. Prior to screening data for artifact for each second using the thresholds described below, raw data for the entire minute were filtered for line noise and any ECG artifact was subtracted from each channel. A 16,000 point finite-impulse response 4 Hz wide notch filter was applied at the line noise frequency and its first harmonic (60 and 120 Hz, or 50 and 100 Hz, depending on the site), with 36 to 100 dB power reduction within the notches. ECG artifact was removed from each channel using a recently developed method that mimics the procedure for ballistocardiogram removal from EEG recorded during MRI[6]. Ballistocardiogram artifact is removed by using a simultaneously recorded ECG signal to precisely identify the times of each R wave peak, and then to signal average the EEG over small windows centered on those times, deriving a channel-specific template that is then subtracted from each channel.

EEG power spectra were then computed for 60-second epochs using the Welch method, averaging over fast Fourier transforms (FFTs) taken each second.[7] Data were demeaned and a Hanning window was applied prior to computing the FFT for each second. To determine the leads and times contaminated by movement-related or other sources of electrical artifact, we applied multiple criteria on a second by second basis to data from each lead. Criteria were as follows: standard deviation of voltage less than 50 μV and greater than 0.001 μV; sample-to-sample change less than 50 μV; absolute value of voltage less than 300 μV; log-log spectral slope of raw data between 20 and 120 Hz less than -0.1 (to screen for muscle artifact). If more than 5 leads had artifact during any one second, that second was excluded. Remaining data were re-referenced to the average over all leads at each sample. Finally, minute by minute power was the average of the squared FFT's over the accepted seconds, requiring at least 30 acceptable seconds per minute for each lead as a minimum inclusion threshold. A natural log was then applied.

Respiration and Sleep State Coding Alignment with EEG. Detailed methods for sleep state, respiration, and heart rate recordings were published previously.[8] For each minute, the variance of the instantaneous breathing rate was used to quantitatively determine sleep state.[9] Minute by minute EEG power was aligned with simultaneous sleep state codes and averaged over REM sleep i.e. active sleep (AS) and non-REM sleep i.e. quiet sleep (QS) minutes within each study.

Missing Data Imputation. For all pregnant women in the Safe Passage Study, daily drinking and smoking data were collected for the last reported drinking day and 30 days prior. For estimates of alcohol consumption, out of 3.2 million person-days of observation, data were missing for 0.36 million (11.4%) days. We excluded subjects with more than 200 days missing and subjects who did not have any alcohol consumption data in the first trimester from the imputation analyses. For estimates of smoking, out of 0.47 million person-weeks, data were missing for 0.13 person-weeks (27.7%). We classified smoking status accurately in 93% of randomly deleted segments, and , for 86% segments imputed data were within +/-1 cigarette/day of the actual. Missing exposure data was imputed using a nonparametric machine learning algorithm called the k nearest neighbor method (kNN).

Alcohol and Smoking Cluster Analysis. In order to discern relationships between different patterns of PAE and PTE during pregnancy on newborn brain activity, we utilized clustering techniques to characterize maternal drinking and smoking behaviors. We have previously published the cluster analysis of alcohol consumption during pregnancy in the Safe Passage Study[1]. In brief, the R-package **clValid**[2] was utilized with an internal validation so the authors could simultaneously evaluate several clustering methods to aid in the authors' determination the most appropriate method and number of clusters based on diverse figures of merit, such as connectivity measures, the Dunn index, and silhouette measures. Based on these metrics and human decision making, finite mixture models were implemented using the R-package **mclust**[3] from derived daily alcohol consumption data for 10,279 participants in the Safe Passage Study. We utilized six features for the alcohol cluster analysis: the sum of standard drinks per day in trimesters 1, 2, and 3 and binge drinking events (≥ 4 drinks on a given day) in trimesters 1, 2, and 3. Ten alcohol trajectory groups were previously defined with one group consisting of non-drinkers (n=5,915)[4]. Hierarchical clustering was implemented using the R-packages **hclust**[5] and **agnes**[2] for 10,941 participants with available smoking information to define smoking cluster groups from four features:

© 2020 Shuffrey LC et al. *JAMA Network Open.*

average cigarettes smoked per week in trimesters 1, 2, and 3, in addition to a quit smoking variable which was defined as < 1 average cigarettes per week in trimesters 2 and 3. The distance metric was Euclidean and linkage method was Ward's minimum variance method. Women having a total trimester-level average cigarette consumption <1 had that corresponding value set to zero. For the remaining 5,155 participants, any outliers for each feature were winsorized and assigned with a value equal to the *Mean* + (4*SD). Lastly, features utilized for the cluster analysis were z-scored to obtain a zero-mean and unitary-variance. We defined five smoking trajectory groups with one group consisting of non-smokers (n=5,786). For some alcohol and smoking exposure cluster cross-tabulations, there were small N's which also had EEG data (n=<10). Therefore, in the present analysis we collapsed the PASS alcohol and smoking cluster groups.

Summary Demographic Information.

Measuring covariates: Maternal-infant charts were abstracted to obtain maternal age at delivery, gestational age at birth, delivery mode, and biological sex. Maternal race and ethnicity, marital status, education, and self-reported recreational drug use were acquired through Safe Passage Study specific questionnaires. The infant's age at the EEG study was determined by their gestational age at birth and postnatal age (in hours) at the time of the EEG study. All participants in the present analysis (n=1,739) had complete data for covariates.

Sources of bias. To account for confounding variables, we excluded subjects if they were born earlier than 37 weeks, later than 41 weeks, twins, or had prenatal exposure to psychiatric medications *in-utero*. In addition, we adjusted for the known confounder postnatal age by utilizing standardized residuals of EEG power and adjusted for gestational age at birth, sex, clinical site, and recreational drug use in the model. To account for bias originating from exposure measurement error, we imputed missing data using a non-parametric algorithm before clustering them into exposure groups

Eligibility Criteria. A total of 11,892 mother-fetal dyads were consented to the Safe Passage Study. After exclusions for missing exposure data (n=1,613), infants with no EEG recording at birth (n=7,375), infants with insufficient EEG data due to equipment failure, poor quality, missing sleep state, or awake sleep state (n=618), preterm birth (n=310), postterm birth (n=180), twins (n=11), and maternal psychiatric use during pregnancy (n=36), a total of 1,739 participants were included in the present analysis (see flowchart below).

© 2020 Shuffrey LC et al. *JAMA Network Open.*

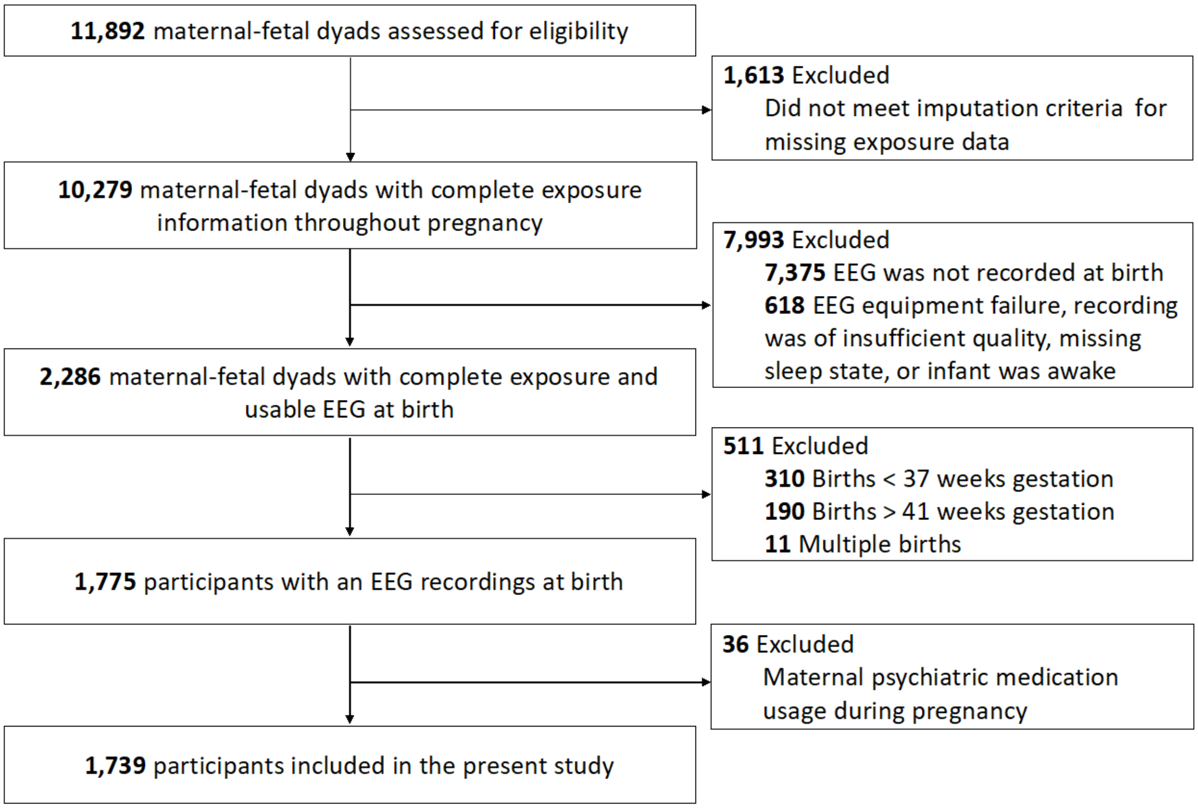

© 2020 Shuffrey LC et al. *JAMA Network Open.*

eTable 1. EEG Frequency Bin to Frequency Name Reference

EEG Frequency Bins	EEG Frequency Name
1 – 3 Hz	Delta (δ)
4 – 6 Hz	Theta (θ)
7 – 9 Hz	Low (Infant) Alpha (α)
10 – 12 Hz	High (Adult) Alpha (α)
13 – 15 Hz, 16 – 18 Hz, 19 – 21 Hz; 22 – 24 Hz,	Beta (β)
25 – 27 Hz, 28 – 30 Hz, 31 – 33 Hz, 34 – 36 Hz	Low Gamma (γ)
37 – 39 Hz, 40 – 42 Hz, 43 – 45 Hz	Gamma (γ)

© 2020 Shuffrey LC et al. *JAMA Network Open.*

eTable 2. Alcohol Clusters. Alcohol Clusters and sample sizes within each collapsed cluster. Also shown are the median of standard drinks per trimester ± the interquartile range, and the median of binge events (≥ 4 drinks per day) for each cluster group ± the interquartile range for participants in the analyses of EEG (total n=1,739)

		Median of Standard Drinks per Trimester ± Interquartile Range			***Median of Binge Events per Trimester ± Interquartile Range***			***Median of Standard Drinks ± Interquartile Range***	***Median of Binge Events ± Interquartile Range***
**Alcohol Cluster Group**	**Sample Size**	**Trimester 1 Alcohol**	**Trimester 2 Alcohol**	**Trimester 3 Alcohol**	**Trimester 1 Binge Events**	**Trimester 2 Binge Events**	**Trimester 3 Binge Events**	**Total Alcohol**	**Total Binge Events**
No Alcohol	778	0.00 ± 0.00	0.00 ± 0.00	0.00 ± 0.00	0.00 ± 0.00	0.00 ± 0.00	0.00 ± 0.00	0.00 ± 0.00	0.00 ± 0.00
Low Continuous Alcohol	178	0.00 ± 3.03	3.35 ± 4.72	0.00 ± 1.39	0.00 ± 0.00	0.00 ± 1.00	0.00 ± 0.00	5.58 ± 5.85	0.00 ± .1.00
Quit Early Alcohol	482	6.13 ± 8.27	0.00 ± 0.00	0.00 ± 0.00	1.00 ± 1.00	0.00 ± 0.00	0.00 ± 0.00	6.14 ± 8.61	1.00 ± 3.00
Moderate or High Continuous Alcohol	301	16.45 ± 48.52	7.05 ± 19.87	3.10 ± 9.24	1.00 ± 5.00	1.00 ± 2.00	0.00 ± 1.00	38.19 ± 51.03	4.00 ± 5.00

© 2020 Shuffrey LC et al. *JAMA Network Open.*

eTable 3. Smoking Clusters. Smoking Cluster Groups and sample sizes within each cluster. Also shown are the median of average cigarettes smoked per trimester ± the interquartile range for each cluster group in the analyses of EEG (total n=1,739)

		Median of Average Cigarettes per Trimester ± Interquartile Range			***Median of Cigarettes ± Interquartile Range***
**Smoking Cluster Group**	**Sample Size**	**Trimester 1 Cigarettes**	**Trimester 2 Cigarettes**	**Trimester 3 Cigarettes**	**Total Cigarettes**
No Smoking	859	0.00 ± 0.00	0.00 ± 0.00	0.00 ± 0.00	0.00 ± 0.00
Low Continuous or Quit Early Smoking	580	14.93 ± 14.93	14.46 ± 11.33	13.05 ± 19.65	43.45 ± 48.27
Moderate or High Continuous Smoking	300	40.71 ± 23.78	43.21 ± 23.84	38.43 ± 21.90	120.77 ± 56.89

© 2020 Shuffrey LC et al. *JAMA Network Open.*

eTable 4. Estimated marginal means for the main effect of alcohol on temporal Theta and Alpha EEG power in active sleep.
[a] *Covariate adjustments in the model include sex, gestational age at birth, recreational drug exposure, and clinical site.*

Brain Region and Frequency Bin	Alcohol Cluster Group	Estimated Marginal Mean	Difference from the reference group	Standard Error	95% CI	
					Lower Bound	Upper Bound
Right Temporal 4 – 6 Hz	No Alcohol	-.116[a]	Reference	0.045	-0.203	-0.028
	Low Continuous Alcohol	.008[a]	106.89 %	0.077	-0.143	0.159
	Quit Alcohol Early	.129[a]	211.207 %	0.059	0.013	0.244
	Moderate or High Continuous Alcohol	.129[a]	211.207 %	0.064	0.036	0.288
Left Temporal 4 – 6 Hz	No Alcohol	-.139[a]	Reference	0.045	-0.226	-0.051
	Low Continuous Alcohol	.066[a]	147.482 %	0.077	-0.085	0.218
	Quit Alcohol Early	.134[a]	196.403 %	0.059	0.019	0.25
	Moderate or High Continuous Alcohol	.138[a]	199.281 %	0.064	0.013	0.264
Left Temporal 7 – 9 Hz	No Alcohol	-.143[a]	Reference	0.045	-0.231	-0.055
	Low Continuous Alcohol	.077[a]	153.846 %	0.078	-0.075	0.229
	Quit Alcohol Early	.109[a]	176. 224 %	0.059	-0.007	0.225
	Moderate or High Continuous Alcohol	.130[a]	190.909 %	0.064	0.004	0.256

© 2020 Shuffrey LC et al. *JAMA Network Open.*

eTable 5. Pairwise comparisons for the main effect of alcohol compared to no prenatal exposure to alcohol.

Alcohol Cluster Group		Mean Difference from No Alcohol Exposure	Std Error	p-value	95 % Confidence Interval	
					Lower Bound	Upper Bound
**4 – 6 Hz Right Temporal EEG Power**						
**No Alcohol**	Low Continuous Alcohol	-.123	0.089	0.163	-0.297	0.050
	Quit Alcohol Early	-.244	0.074	0.001**	-0.390	-0.099
	Moderate or High Continuous Alcohol	-.278	0.078	0.000***	-0.431	-0.125
**4– 6 Hz Left Temporal EEG Power**						
**No Alcohol**	Low Continuous Alcohol	-.205	0.089	0.021*	-0.379	-0.031
	Quit Alcohol Early	-.273	0.074	0.000***	-0.419	-0.127
	Moderate or High Continuous Alcohol	-.277	0.078	0.000***	-0.430	-0.124
**7– 9 Hz Left Temporal EEG Power**						
**No Alcohol**	Low Continuous Alcohol	-.220	0.089	0.014*	-0.395	-0.045
	Quit Alcohol Early	-.252	0.075	0.001**	-0.398	-0.106
	High Continuous Alcohol	-.273	0.078	0.001**	-0.427	-0.119

© 2020 Shuffrey LC et al. *JAMA Network Open.*

eTable 6. Estimated marginal means for the main effect of smoking on right central EEG power in active sleep.
[a] *Covariate adjustments in the model include sex, gestational age at birth, recreational drug exposure, and clinical site.*

Brain Region and Frequency Bin	Smoking Cluster Group	Estimated Marginal Mean	Difference from the reference group	Standard Error	95% CI	
					Lower Bound	Upper Bound
Right Central 19 - 21 Hz	No Smoking	-0.065	Reference	0.055	-0.172	0.042
	Low Continuous or Quit Smoking	0.099	252.308 %	0.046	0.007	0.190
	Moderate or High Continuous Smoking	-0.151	- 132.308 %	0.066	-0.28	-0.022
Right Central 22 - 24 Hz	No Smoking	-0.065	Reference	0.055	-0.173	0.042
	Low Continuous or Quit Smoking	0.097	249.231 %	0.047	0.006	0.188
	Moderate or High Continuous Smoking	-0.163	- 150.769 %	0.066	-0.292	-0.033
Right Central 25 - 27 Hz	No Smoking	-0.085	Reference	0.055	-0.192	0.023
	Low Continuous Alcohol	0.093	209.412 %	0.047	0.002	0.184
	Moderate or High Continuous Smoking	-0.127	- 49.4112 %	0.066	-0.256	0.002
Right Central 28 - 30 Hz	No Smoking	-0.091	Reference	0.055	-0.199	0.017
	Low Continuous or Quit Smoking	0.115	226.3774 %	0.047	0.024	0.206
	Moderate or High Continuous Smoking	-0.136	- 49.451 %	0.066	-0.266	-0.006
Right Central 31 - 33 Hz	No Smoking	-0.088	Reference	0.055	-0.196	0.019
	Low Continuous or Quit Smoking	0.119	235. 227 %	0.047	0.028	0.211
	Moderate or High Continuous Smoking	-0.134	- 52.272 %	0.066	-0.264	-0.005
Right Central 34 - 36 Hz	No Smoking	-0.090	Reference	0.055	-0.198	0.018
	Low Continuous or Quit Smoking	0.123	236.667 %	0.047	0.032	0.215
	Moderate or High Continuous Smoking	-0.116	- 28.889 %	0.066	-0.246	0.014

© 2020 Shuffrey LC et al. *JAMA Network Open.*

eTable 7. Pairwise comparisons for the effect of smoking on Beta (19 – 24 Hz) right central EEG power in active sleep

Smoking Cluster Group		Mean Difference	Std Error	p-value	95 % Confidence Interval	
					**Lower Bound**	**Upper Bound**
**19 - 21 Hz Right Central EEG Power**						
**No Smoking**	Low Continuous or Quit Smoking	-0.164	0.073	0.025*	-0.306	-0.021
	Moderate or High Continuous Smoking	0.086	0.087	0.323	-0.085	0.257
**Low Continuous or Quit**	No Smoking	0.164	0.073	0.025*	0.021	0.306
	Moderate or High Continuous Smoking	0.250	0.079	0.002**	0.096	0.404
**Moderate or High Continuous Smoking**	No Smoking	-0.086	0.087	0.323	-0.257	0.085
	Low Continuous or Quit Smoking	-0.250	0.079	0.002**	-0.404	-0.096
**22 - 24 Hz Right Central EEG Power**						
**No Smoking**	Low Continuous or Quit Smoking	-0.162	0.073	0.027*	-0.305	-0.019
	Moderate or High Continuous Smoking	0.098	0.087	0.263	-0.073	0.269
**Low Continuous or Quit**	No Smoking	0.162	0.073	0.027*	0.019	0.305
	Moderate or High Continuous Smoking	0.260	0.079	0.001**	0.105	0.414
**Moderate or High Continuous Smoking**	No Smoking	-0.098	0.087	0.263	-0.269	0.073
	Low Continuous or Quit Smoking	-0.260	0.079	0.001**	-0.414	-0.105

© 2020 Shuffrey LC et al. *JAMA Network Open.*

eTable 8. Pairwise comparisons for the effect of smoking on low Gamma (25 – 37 Hz) right central EEG power in active sleep

Smoking Cluster Group		Mean Difference	Std Error	p-value	95 % Confidence Interval	
					**Lower Bound**	**Upper Bound**
**25 - 27 Hz Right Central EEG Power**						
**No Smoking**	Low Continuous or Quit Smoking	-0.177	0.073	0.015*	-0.321	-0.034
	Moderate or High Continuous Smoking	0.042	0.087	0.627	-0.129	0.214
**Low Continuous or Quit**	No Smoking	0.177	0.073	0.015*	0.034	0.321
	Moderate or High Continuous Smoking	0.220	0.079	0.005**	0.065	0.374
**Moderate or High Continuous Smoking**	No Smoking	-0.042	0.087	0.627	-0.214	0.129
	Low Continuous or Quit Smoking	-0.22	0.079	0.005**	-0.374	-0.065
**28 - 30 Hz Right Central EEG Power**						
**No Smoking**	Low Continuous or Quit Smoking	-0.206	0.073	0.005**	-0.349	-0.063
	Moderate or High Continuous Smoking	0.045	0.087	0.607	-0.126	0.216
**Low Continuous or Quit**	No Smoking	0.206	0.073	0.005**	0.063	0.349
	Moderate or High Continuous Smoking	0.251	0.079	0.001**	0.096	0.406
**Moderate or High Continuous Smoking**	No Smoking	-0.045	0.087	0.607	-0.216	0.126
	Low Continuous or Quit Smoking	-0.251	0.079	0.001**	-0.406	-0.096
**31 - 33 Hz Right Central EEG Power**						
**No Smoking**	Low Continuous or Quit Smoking	-0.208	0.073	0.005**	-0.351	-0.064
	Moderate or High Continuous Smoking	0.046	0.087	0.599	-0.125	0.217
**Low Continuous or Quit**	No Smoking	0.208	0.073	0.005**	0.064	0.351
	Moderate or High Continuous Smoking	0.254	0.079	0.001**	0.099	0.408
**Moderate or High Continuous Smoking**	No Smoking	-0.046	0.087	0.599	-0.217	0.125
	Low Continuous or Quit Smoking	-0.254	0.079	0.001**	-0.408	-0.099
**34 - 36 Hz Right Central EEG Power**						

© 2020 Shuffrey LC et al. *JAMA Network Open.*

**No Smoking**	Low Continuous or Quit Smoking	-0.213	0.073	0.004**	-0.356	-0.07
	Moderate or High Continuous Smoking	0.026	0.087	0.765	-0.145	0.197
**Low Continuous or Quit**	No Smoking	0.213	0.073	0.004**	0.070	0.356
	Moderate or High Continuous Smoking	0.239	0.079	0.002**	0.084	0.394
**Moderate or High Continuous Smoking**	No Smoking	-0.026	0.087	0.765	-0.197	0.145
	Low Continuous or Quit Smoking	-0.239	0.079	0.002**	-0.394	-0.084

© 2020 Shuffrey LC et al. *JAMA Network Open.*

eTable 9. Estimated marginal means for the main effect of smoking on right parietal EEG power in active sleep

Brain Region and Frequency Bin	Smoking Cluster Group	Estimated Marginal Mean	Difference from the reference group	Standard Error	95% CI	
					Lower Bound	Upper Bound
Right Parietal 28 - 30 Hz	No Smoking	-0.105	Reference	0.055	-0.212	0.002
	Low Continuous Alcohol	0.113	207.619 %	0.046	0.023	0.204
	Moderate or High Continuous Smoking	-0.061	41.905 %	0.065	-0.189	0.067
Right Parietal 31 - 33 Hz	No Smoking	-0.111	Reference	0.055	-0.219	-0.004
	Low Continuous Alcohol	0.117	205.405 %	0.046	0.026	0.207
	Moderate or High Continuous Smoking	-0.061	45.045 %	0.065	-0.188	0.067
Right Parietal 34 - 36 Hz	No Smoking	-0.115	Reference	0.055	-0.222	-0.008
	Low Continuous Alcohol	0.117	201.739 %	0.046	0.026	0.207
	Moderate or High Continuous Smoking	-0.056	51.304 %	0.065	-0.184	0.071
Right Parietal 37 – 39 Hz	No Smoking	-0.114	Reference	0.055	-0.221	-0.007
	Low Continuous Alcohol	0.109	195.614 %	0.046	0.019	0.199
	Moderate or High Continuous Smoking	-0.058	49.123 %	0.065	-0.185	0.07
Right Parietal 43 – 45 Hz	No Smoking	-0.132	Reference	0.055	-0.239	-0.025
	Low Continuous Alcohol	0.105	179.545 %	0.046	0.014	0.195
	Moderate or High Continuous Smoking	-0.056	57.578 %	0.065	-0.183	0.072

© 2020 Shuffrey LC et al. *JAMA Network Open.*

eTable 10. Pairwise comparisons for the effect of smoking on low Gamma (28 – 36 Hz) right parietal EEG power in active sleep

Smoking Cluster Group		Mean Difference	Std Error	p-value	95 % Confidence Interval	
					**Lower Bound**	**Upper Bound**
**28 – 30 Hz Right Parietal EEG Power**						
**No Smoking**	Low Continuous or Quit Smoking	-0.218	0.073	0.003**	-0.361	-0.076
	Moderate or High Continuous Smoking	-0.044	0.087	0.610	-0.214	0.126
**Low Continuous or Quit**	No Smoking	0.218	0.073	0.003**	0.076	0.361
	Moderate or High Continuous Smoking	0.174	0.078	0.025*	0.022	0.327
**Moderate or High Continuous Smoking**	No Smoking	0.044	0.087	0.610	-0.126	0.214
	Low Continuous or Quit Smoking	-0.174	0.078	0.025*	-0.327	-0.022
**31 – 33 Hz Right Parietal EEG Power**						
**No Smoking**	Low Continuous or Quit Smoking	-0.228	0.073	0.002**	-0.371	-0.086
	Moderate or High Continuous Smoking	-0.051	0.087	0.557	-0.220	0.119
**Low Continuous or Quit**	No Smoking	0.228	0.073	0.002**	0.086	0.371
	Moderate or High Continuous Smoking	0.178	0.078	0.023*	0.025	0.330
**Moderate or High Continuous Smoking**	No Smoking	0.051	0.087	0.557	-0.119	0.220
	Low Continuous or Quit Smoking	-0.178	0.078	0.023*	-0.330	-0.025
**34 - 36 Right Parietal EEG Power**						
**No Smoking**	Low Continuous or Quit Smoking	-0.232	0.073	0.001**	-0.374	-0.090
	Moderate or High Continuous Smoking	-0.059	0.086	0.494	-0.229	0.110
**Low Continuous or Quit**	No Smoking	0.232	0.073	0.001**	0.090	0.374
	Moderate or High Continuous Smoking	0.173	0.078	0.026*	0.020	0.325
**Moderate or High Continuous Smoking**	No Smoking	0.059	0.086	0.494	-0.110	0.229
	Low Continuous or Quit Smoking	-0.173	0.078	0.026*	-0.325	-0.020

© 2020 Shuffrey LC et al. *JAMA Network Open.*

eTable 11. Pairwise comparisons for the effect of smoking on Gamma (37 – 39; 43 – 45 Hz) right parietal EEG power in active sleep

Smoking Cluster Group		Mean Difference	Std Error	p-value	95 % Confidence Interval	
					**Lower Bound**	**Upper Bound**
**37 – 39 Hz Right Parietal EEG Power**						
**No Smoking**	Low Continuous or Quit Smoking	-0.223	0.072	0.002**	-0.365	-0.080
	Moderate or High Continuous Smoking	-0.056	0.086	0.516	-0.225	0.113
**Low Continuous or Quit**	No Smoking	0.223	0.072	0.002**	0.080	0.365
	Moderate or High Continuous Smoking	0.167	0.078	0.032*	0.014	0.319
**Moderate or High Continuous Smoking**	No Smoking	0.056	0.086	0.516	-0.113	0.225
	Low Continuous or Quit Smoking	-0.167	0.078	0.032*	-0.319	-0.014
**43 – 45 Hz Right Parietal EEG Power**						
**No Smoking**	Low Continuous or Quit Smoking	-0.236	0.073	0.001**	-0.379	-0.094
	Moderate or High Continuous Smoking	-0.076	0.086	0.379	-0.245	0.093
**Low Continuous or Quit**	No Smoking	0.236	0.073	0.001**	0.094	0.379
	Moderate or High Continuous Smoking	0.161	0.078	0.039*	0.008	0.313
**Moderate or High Continuous Smoking**	No Smoking	0.076	0.086	0.379	-0.093	0.245
	Low Continuous or Quit Smoking	-0.161	0.078	0.039*	-0.313	-0.008

© 2020 Shuffrey LC et al. *JAMA Network Open.*

eTable 12. 4-level Collapsed Smoking Cluster. Groups and sample sizes within each cluster, the median of average cigarettes smoking per trimester ± the interquartile range for participants included in the analyses of EEG.

		Median of Average Cigarettes per Trimester ± Interquartile Range			***Median of Cigarettes ± Interquartile Range***
**Smoking Cluster Group**	**Sample Size**	**Trimester 1 Cigarettes**	**Trimester 2 Cigarettes**	**Trimester 3 Cigarettes**	**Total Cigarettes**
**No Smoking**	859	0.00 ± 0.00	0.00 ± 0.00	0.00 ± 0.00	0.00 ± 0.00
**Quit Early Smoking**	66	6.95 ± 13.21	0.00 ± 0.00	0.00 ± 0.00	6.96 ± 13.33
**Low Continuous Smoking**	514	15.52 ± 14.21	16.60 ± 17.60	15.59 ± 17.52	50.30 ± 43.49
Moderate or High Continuous Smoking	300	40.71 ± 23.78	43.21 ± 23.84	38.43 ± 21.90	120.77 ± 56.89

© 2020 Shuffrey LC et al. *JAMA Network Open.*

eTable 13. Estimated marginal means for the main effect of smoking using a 4-level smoking variable on right central EEG power in active sleep. [a] *Covariate adjustments in the model include sex, gestational age at birth, prenatal alcohol exposure, recreational drug exposure, and clinical site.*

Brain Region and Frequency Bin	Smoking Cluster Group	Estimated Marginal Mean	Difference from the reference group	Standard Error	95% CI	
					Lower Bound	Upper Bound
Right Central 19 - 21 Hz	No Smoking	.030[a]	Reference	0.037	-0.043	0.103
	Quit Smoking	.117[a]	290.000 %	0.132	-0.142	0.376
	Low Continuous Smoking	.045[a]	250.000 %	0.047	-0.046	0.137
	Moderate or High Continuous Smoking	-.151[a]	-603.333 %	0.062	-0.272	-0.029
Right Central 22 - 24 Hz	No Smoking	.022[a]	Reference	0.037	-0.051	0.096
	Quit Smoking	.144[a]	554.545 %	0.132	-0.115	0.403
	Low Continuous Smoking	.052[a]	136.363 %	0.047	-0.040	0.144
	Moderate or High Continuous Smoking	-.146[a]	- 763.636 %	0.062	-0.268	-0.025
Right Central 25 - 27 Hz	No Smoking	.005[a]	Reference	0.037	-0.068	0.079
	Quit Smoking	.188[a]	3660.000 %	0.132	-0.071	0.447
	Low Continuous Smoking	.048[a]	860.000 %	0.047	-0.043	0.140
	Moderate or High Continuous Smoking	-.109[a]	- 2280.000 %	0.062	-0.230	0.013
Right Central 28 - 30 Hz	No Smoking	.004[a]	Reference	0.037	-0.069	0.078
	Quit Smoking	.182[a]	4450.000 %	0.132	-0.077	0.442
	Low Continuous Smoking	.068[a]	1600.000 %	0.047	-0.024	0.16
	Moderate or High Continuous Smoking	-.135[a]	- 3475.000 %	0.062	-0.257	-0.014
Right Central 31 - 33 Hz	No Smoking	.000[a]	Reference	0.037	-0.073	0.074
	Quit Smoking	.185[a]	184,900.000 %	0.132	-0.075	0.444
	Low Continuous Smoking	.072[a]	71,900.000 %	0.047	-0.020	0.163
	Moderate or High Continuous Smoking	-.143[a]	142,900.000 %	0.062	-0.265	-0.021
	No Smoking	-.002[a]	Reference	0.037	-0.075	0.071

© 2020 Shuffrey LC et al. *JAMA Network Open.*

Right Central 34 - 36 Hz	Quit Smoking	.216[a]	10,900.000 %	0.132	-0.043	0.476
	Low Continuous Smoking	.068[a]	3,500.000 %	0.047	-0.024	0.159
	Moderate or High Continuous Smoking	-.130[a]	- 6,400.000 %	0.062	-0.252	-0.009

© 2020 Shuffrey LC et al. *JAMA Network Open.*

eTable 14. Pairwise comparisons for the effect of smoking on Beta (19 - 24 Hz) right central EEG power in active sleep

Smoking Cluster Group		Mean Difference	Std Error	p-value	95 % Confidence Interval	
					**Lower Bound**	**Upper Bound**
**19 - 21 Hz Right Central EEG Power**						
**No Smoking**	Quit Smoking	-0.087	0.136	0.521	-0.354	0.180
	Low Continuous Smoking	-0.016	0.062	0.802	-0.137	0.106
	Moderate or High Continuous Smoking	0.180	0.075	0.016*	0.034	0.327
**Quit Smoking**	No Smoking	0.087	0.136	0.521	-0.18	0.354
	Low Continuous Smoking	0.072	0.141	0.610	-0.205	0.348
	Moderate or High Continuous Smoking	0.268	0.147	0.069	-0.021	0.556
**Low Continuous Smoking**	No Smoking	0.016	0.062	0.802	-0.106	0.137
	Quit Smoking	-0.072	0.141	0.610	-0.348	0.205
	Moderate or High Continuous Smoking	0.196	0.075	0.009**	0.049	0.343
**Moderate or High Continuous Smoking**	No Smoking	-0.180	0.075	0.016*	-0.327	-0.034
	Quit Smoking	-0.268	0.147	0.069	-0.556	0.021
	Low Continuous Smoking	-0.196	0.075	0.009**	-0.343	-0.049
**22-24 Hz Right Central EEG Power**						
**No Smoking**	Quit Smoking	-0.122	0.136	0.373	-0.389	0.146
	Low Continuous Smoking	-0.030	0.062	0.634	-0.151	0.092
	Moderate or High Continuous Smoking	0.169	0.075	0.024*	0.022	0.316
**Quit Smoking**	No Smoking	0.122	0.136	0.373	-0.146	0.389
	Low Continuous Smoking	0.092	0.141	0.515	-0.185	0.369
	Moderate or High Continuous Smoking	0.290	0.147	0.049*	0.001	0.579
**Low Continuous Smoking**	No Smoking	0.030	0.062	0.634	-0.092	0.151
	Quit Smoking	-0.092	0.141	0.515	-0.369	0.185
	Moderate or High Continuous Smoking	0.198	0.075	0.008**	0.051	0.346
	No Smoking	-0.169	0.075	0.024*	-0.316	-0.022

© 2020 Shuffrey LC et al. *JAMA Network Open.*

**Moderate or High Continuous Smoking**	Quit Smoking	-0.290	0.147	0.049*	-0.579	-0.001
	Low Continuous Smoking	-0.198	0.075	0.008**	-0.346	-0.051

© 2020 Shuffrey LC et al. *JAMA Network Open.*

eTable 15. Pairwise comparisons for the effect of smoking on low Gamma (25 - 36 Hz) right central EEG power in active sleep

Smoking Cluster Group		Mean Difference	Std Error	p-value	95 % Confidence Interval	
					**Lower Bound**	**Upper Bound**
**25 - 27 Hz Right Central EEG Power**						
No Smoking	Quit Smoking	-0.183	0.136	0.180	-0.451	0.085
	Low Continuous Smoking	-0.043	0.062	0.486	-0.165	0.079
	Moderate or High Continuous Smoking	0.114	0.075	0.129	-0.033	0.261
Quit Smoking	No Smoking	0.183	0.136	0.180	-0.085	0.451
	Low Continuous Smoking	0.14	0.141	0.323	-0.137	0.417
	Moderate or High Continuous Smoking	0.297	0.147	0.044*	0.008	0.586
Low Continuous Smoking	No Smoking	0.043	0.062	0.486	-0.079	0.165
	Quit Smoking	-0.14	0.141	0.323	-0.417	0.137
	Moderate or High Continuous Smoking	0.157	0.075	0.037*	0.009	0.305
Moderate or High Continuous Smoking	No Smoking	-0.114	0.075	0.129	-0.261	0.033
	Quit Smoking	-0.297	0.147	0.044*	-0.586	-0.008
	Low Continuous Smoking	-0.157	0.075	0.037*	-0.305	-0.009
**28 - 30 Hz Right Central EEG Power**						
No Smoking	Quit Smoking	-0.178	0.136	0.192	-0.446	0.090
	Low Continuous Smoking	-0.064	0.062	0.304	-0.186	0.058
	Moderate or High Continuous Smoking	0.14	0.075	0.063	-0.007	0.287
Quit Smoking	No Smoking	0.178	0.136	0.192	-0.090	0.446
	Low Continuous Smoking	0.114	0.141	0.419	-0.163	0.392
	Moderate or High Continuous Smoking	0.318	0.148	0.031*	0.028	0.607
Low Continuous Smoking	No Smoking	0.064	0.062	0.304	-0.058	0.186
	Quit Smoking	-0.114	0.141	0.419	-0.392	0.163
	Moderate or High Continuous Smoking	0.203	0.075	0.007**	0.056	0.351
Moderate or High Continuous Smoking	No Smoking	-0.14	0.075	0.063	-0.287	0.007
	Quit Smoking	-0.318	0.148	0.031*	-0.607	-0.028
	Low Continuous Smoking	-0.203	0.075	0.007**	-0.351	-0.056
**31 - 33 Hz Right Central EEG Power**						
No Smoking	Quit Smoking	-0.184	0.137	0.177	-0.452	0.084
	Low Continuous Smoking	-0.071	0.062	0.250	-0.193	0.050

© 2020 Shuffrey LC et al. *JAMA Network Open.*

	Moderate or High Continuous Smoking	0.143	0.075	0.057	-0.004	0.290
Quit Smoking	No Smoking	0.184	0.137	0.177	-0.084	0.452
	Low Continuous Smoking	0.113	0.142	0.425	-0.165	0.390
	Moderate or High Continuous Smoking	0.327	0.148	0.027*	0.038	0.617
Low Continuous Smoking	No Smoking	0.071	0.062	0.250	-0.050	0.193
	Quit Smoking	-0.113	0.142	0.425	-0.390	0.165
	Moderate or High Continuous Smoking	0.215	0.075	0.004**	0.067	0.362
Moderate or High Continuous Smoking	No Smoking	-0.143	0.075	0.057	-0.290	0.004
	Quit Smoking	-0.327	0.148	0.027*	-0.617	-0.038
	Low Continuous Smoking	-0.215	0.075	0.004**	-0.362	-0.067
**34 - 36 Hz Right Central EEG Power**						
No Smoking	Quit Smoking	-0.218	0.137	0.111	-0.486	0.050
	Low Continuous Smoking	-0.070	0.062	0.262	-0.192	0.052
	Moderate or High Continuous Smoking	0.128	0.075	0.088	-0.019	0.275
Quit Smoking	No Smoking	0.218	0.137	0.111	-0.050	0.486
	Low Continuous Smoking	0.148	0.142	0.294	-0.129	0.426
	Moderate or High Continuous Smoking	0.346	0.148	0.019*	0.057	0.636
Low Continuous Smoking	No Smoking	0.070	0.062	0.262	-0.052	0.192
	Quit Smoking	-0.148	0.142	0.294	-0.426	0.129
	Moderate or High Continuous Smoking	0.198	0.075	0.009**	0.050	0.346
Moderate or High Continuous Smoking	No Smoking	-0.128	0.075	0.088	-0.275	0.019
	Quit Smoking	-.0346	0.148	0.019*	-0.636	-0.057
	Low Continuous Smoking	-0.198	0.075	0.009**	-0.346	-0.050

© 2020 Shuffrey LC et al. *JAMA Network Open.*

eTable 16. Estimated marginal means for the main effect of smoking using a 4-level smoking variable on right parietal EEG power in active sleep. [a] *Covariate adjustments in the model include sex, gestational age at birth, prenatal alcohol exposure, recreational drug exposure, and clinical site.*

Brain Region and Frequency Bin	Smoking Cluster Group	Estimated Marginal Mean	Difference from the reference group	Standard Error	95% CI	
					Lower Bound	Upper Bound
Right Parietal 28 – 30 Hz	No Smoking	-.025[a]	Reference	0.037	-0.098	0.047
	Quit Smoking	.065[a]	360.000 %	0.129	-0.187	0.318
	Low Continuous Smoking	.101[a]	504.000 %	0.046	0.010	0.192
	Moderate or High Continuous Smoking	-.074[a]	-196.000 %	0.061	-0.194	0.045
Right Parietal 31 – 33 Hz	No Smoking	-.030[a]	Reference	0.037	-0.103	0.043
	Quit Smoking	.073[a]	343.333 %	0.129	-0.180	0.325
	Low Continuous Smoking	.104[a]	446.667 %	0.046	0.013	0.195
	Moderate or High Continuous Smoking	-.077[a]	- 156.667	0.061	-0.196	0.042
Right Parietal 34 – 36 Hz	No Smoking	-.030[a]	Reference	0.037	-0.103	0.043
	Quit Smoking	.080[a]	366.667 %	0.129	-0.172	0.332
	Low Continuous Smoking	.104[a]	446.667 %	0.046	0.013	0.195
	Moderate or High Continuous Smoking	-.073[a]	- 143.333 %	0.061	-0.192	0.046
Right Parietal 37 – 39 Hz	No Smoking	-.022[a]	Reference	0.037	-0.094	0.051
	Quit Smoking	.061[a]	377.272 %	0.129	-0.191	0.313
	Low Continuous Smoking	.096[a]	536.363 %	0.046	0.005	0.187
	Moderate or High Continuous Smoking	-.073[a]	- 231.818 %	0.061	-0.192	0.046
Right Parietal 43 – 45 Hz	No Smoking	-.026[a]	Reference	0.037	-0.099	0.047
	Quit Smoking	.167[a]	742.307 %	0.129	-0.085	0.419
	Low Continuous Smoking	.076[a]	392.308 %	0.046	-0.015	0.167
	Moderate or High Continuous Smoking	-.049[a]	-88.462 %	0.061	-0.168	0.070

© 2020 Shuffrey LC et al. *JAMA Network Open.*

eTable 17. Pairwise comparisons for the effect of smoking on low Gamma (28 – 36) right parietal EEG power in active sleep

Smoking Cluster Group		Mean Difference	Std Error	p-value	95 % Confidence Interval	
					**Lower Bound**	**Upper Bound**
**28 – 30 Hz Right Parietal EEG Power**						
No Smoking	Quit Smoking	-0.091	0.133	0.495	-0.352	0.170
	Low Continuous Smoking	-0.126	0.062	0.041*	-0.247	-0.005
	Moderate or High Continuous Smoking	0.049	0.074	0.506	-0.096	0.194
Quit Smoking	No Smoking	0.091	0.133	0.495	-0.170	0.352
	Low Continuous Smoking	-0.035	0.138	0.797	-0.306	0.235
	Moderate or High Continuous Smoking	0.140	0.144	0.331	-0.142	0.422
Low Continuous Smoking	No Smoking	0.126	0.062	0.041*	0.005	0.247
	Quit Smoking	0.035	0.138	0.797	-0.235	0.306
	Moderate or High Continuous Smoking	0.175	0.074	0.018*	0.030	0.321
Moderate or High Continuous Smoking	No Smoking	-0.049	0.074	0.506	-0.194	0.096
	Quit Smoking	-0.140	0.144	0.331	-0.422	0.142
	Low Continuous Smoking	-0.175	0.074	0.018*	-0.321	-0.030
**31 - 33 Hz Right Parietal EEG Power**						
No Smoking	Quit Smoking	-0.103	0.133	0.439	-0.364	0.158
	Low Continuous Smoking	-0.135	0.062	0.029*	-0.256	-0.013
	Moderate or High Continuous Smoking	0.046	0.074	0.530	-0.098	0.191
Quit Smoking	No Smoking	0.103	0.133	0.439	-0.158	0.364
	Low Continuous Smoking	-0.032	0.138	0.819	-0.302	0.239
	Moderate or High Continuous Smoking	0.149	0.144	0.299	-0.133	0.431
Low Continuous Smoking	No Smoking	0.135	0.062	0.029*	0.013	0.256
	Quit Smoking	0.032	0.138	0.819	-0.239	0.302
	Moderate or High Continuous Smoking	0.181	0.074	0.015*	0.036	0.326
Moderate or High Continuous Smoking	No Smoking	-0.046	0.074	0.530	-0.191	0.098
	Quit Smoking	-0.149	0.144	0.299	-0.431	0.133
	Low Continuous Smoking	-0.181	0.074	0.015*	-0.326	-0.036
**34 - 36 Hz Right Parietal EEG Power**						
No Smoking	Quit Smoking	-0.110	0.133	0.407	-0.371	0.151
	Low Continuous Smoking	-0.134	0.062	0.030*	-0.255	-0.013

© 2020 Shuffrey LC et al. *JAMA Network Open.*

	Moderate or High Continuous Smoking	0.043	0.074	0.561	-0.102	0.187
Quit Smoking	No Smoking	0.110	0.133	0.407	-0.151	0.371
	Low Continuous Smoking	-0.024	0.138	0.862	-0.294	0.247
	Moderate or High Continuous Smoking	0.153	0.144	0.287	-0.129	0.435
Low Continuous Smoking	No Smoking	0.134	0.062	0.030*	0.013	0.255
	Quit Smoking	0.024	0.138	0.862	-0.247	0.294
	Moderate or High Continuous Smoking	0.177	0.074	0.017*	0.032	0.322
Moderate or High Continuous Smoking	No Smoking	-0.043	0.074	0.561	-0.187	0.102
	Quit Smoking	-0.153	0.144	0.287	-0.435	0.129
	Low Continuous Smoking	-0.177	0.074	0.017*	-0.322	-0.032

© 2020 Shuffrey LC et al. *JAMA Network Open.*

eTable 18. Pairwise comparisons for the effect of smoking on Gamma (37 – 39; 43 – 25 Hz) right parietal EEG power in active sleep

Smoking Cluster Group		Mean Difference	Std Error	p-value	95 % Confidence Interval	
					**Lower Bound**	**Upper Bound**
**37 - 39 Hz Right Parietal EEG Power**						
No Smoking	Quit Smoking	-0.083	0.133	0.533	-0.343	0.178
	Low Continuous Smoking	-0.118	0.062	0.056	-0.239	0.003
	Moderate or High Continuous Smoking	0.051	0.074	0.488	-0.093	0.195
Quit Smoking	No Smoking	0.083	0.133	0.533	-0.178	0.343
	Low Continuous Smoking	-0.035	0.138	0.799	-0.305	0.235
	Moderate or High Continuous Smoking	0.134	0.144	0.351	-0.148	0.415
Low Continuous Smoking	No Smoking	0.118	0.062	0.056	-0.003	0.239
	Quit Smoking	0.035	0.138	0.799	-0.235	0.305
	Moderate or High Continuous Smoking	0.169*	0.074	0.023*	0.024	0.314
Moderate or High Continuous Smoking	No Smoking	-0.051	0.074	0.488	-0.195	0.093
	Quit Smoking	-0.134	0.144	0.351	-0.415	0.148
	Low Continuous Smoking	-0.169*	0.074	0.023*	-0.314	-0.024
**43 - 45 Hz Right Parietal EEG Power**						
No Smoking	Quit Smoking	-0.192	0.133	0.148	-0.453	0.068
	Low Continuous Smoking	-0.102	0.062	0.099	-0.223	0.019
	Moderate or High Continuous Smoking	0.024	0.074	0.748	-0.121	0.168
Quit Smoking	No Smoking	0.192	0.133	0.148	-0.068	0.453
	Low Continuous Smoking	0.091	0.138	0.511	-0.180	0.361
	Moderate or High Continuous Smoking	0.216	0.144	0.132	-0.066	0.498
Low Continuous Smoking	No Smoking	0.102	0.062	0.099	-0.019	0.223
	Quit Smoking	-0.091	0.138	0.511	-0.361	0.180
	Moderate or High Continuous Smoking	0.126	0.074	0.090	-0.020	0.271
Moderate or High Continuous Smoking	No Smoking	-0.024	0.074	0.748	-0.168	0.121
	Quit Smoking	-0.216	0.144	0.132	-0.498	0.066
	Low Continuous Smoking	-0.126	0.074	0.090	-0.271	0.020

© 2020 Shuffrey LC et al. *JAMA Network Open.*

eReferences.

1. Pini N, Myers MM, Elliott AJ, et al. Cluster Analysis of Alcohol Consumption during Pregnancy in the Safe Passage Study. *Conf Proc IEEE Eng Med Biol Soc.* 2019;2019:1338-1341.
2. Brock G, Datta S, Pihur V, Datta S. clValid: An R package for cluster validation. *Journal of Statistical Software.* 2008;25(4):1-22.
3. Scrucca L, Fop M, Murphy TB, Raftery AE. mclust 5: Clustering, Classification and Density Estimation Using Gaussian Finite Mixture Models. *R J.* 2016;8(1):289-317.
4. Pini N, Shuffrey, LC., Lucchini, M., Sania, A., Nelson, ME., Odendaal, HJ., Fifer, WP., Myers, MM., Elliott, AJ. Characterization of Alcohol Consumption during Pregnancy in the Safe Passage Study. Conf Proc IEEE Eng Med Biol Soc; Berlin, Germany.
5. Langfelder P, Horvath S. Fast R Functions for Robust Correlations and Hierarchical Clustering. *Journal of Statistical Software.* 2012;46(11):1-17.
6. Allen PJ, Polizzi G, Krakow K, Fish DR, Lemieux L. Identification of EEG events in the MR scanner: The problem of pulse artifact and a method for its subtraction. *Neuroimage.* 1998;8(3):229-239.
7. Bendat JS. Citation Classic - Random Data - Analysis and Measurement Procedures. *Cc/Eng Tech Appl Sci.* 1982(25):16-16.
8. Myers MM, Elliott AJ, Odendaal HJ, et al. Cardiorespiratory physiology in the safe passage study: protocol, methods and normative values in unexposed infants. *Acta Paediatr.* 2017;106(8):1260-1272.
9. Isler JR, Thai T, Myers MM, Fifer WP. An automated method for coding sleep states in human infants based on respiratory rate variability. *Developmental Psychobiology.* 2016;58(8):1108-1115.

© 2020 Shuffrey LC et al. *JAMA Network Open.*
